# Vital Carbohydrate and Lipid Metabolites in Serum Involved in Energy Metabolism during Pubertal Molt of Mud Crab (*Scylla paramamosain*)

**DOI:** 10.3390/metabo11100651

**Published:** 2021-09-23

**Authors:** Wen-Feng Li, Shuang Li, Jie Liu, Xiao-Fei Wang, Hui-Yun Chen, Hua Hao, Ke-Jian Wang

**Affiliations:** 1 State Key Laboratory of Marine Environmental Science, College of Ocean & Earth Sciences, Xiamen University, Xiamen 361102, China; liwenfeng2369@163.com (W.-F.L.); lishuanggrace1995@163.com (S.L.); liujie19880209@126.com (J.L.); wxiaofei2021@163.com (X.-F.W.); hychen-2004@163.com (H.-Y.C.); hhao@xmu.edu.cn (H.H.); 2 State-Province Joint Engineering Laboratory of Marine Bioproducts and Technology, College of Ocean & Earth Sciences, Xiamen University, Xiamen 361102, China

**Keywords:** *Scylla paramamosain*, pubertal molt, serum, metabolomics, gas chromatograph, liquid chromatography, mass spectrometry, carbohydrates, lipids

## Abstract

Pubertal molt is a vital stage in the cultivation of mature female crabs in the aquacultural industry of *Scylla paramamosain*. Since fasting occurs during molting, which requires a large supply of energy, internal energy reserves are critical. However, the dynamics of energy supply during pubertal molt is not clear. This study focuses on the variations of carbohydrates and lipids in serum during the pubertal molt of *S. paramamosain* via a metabolomics approach. Eleven lipid or carbohydrate metabolic pathways were significantly influenced postmolt. A remarkable decrease in carbohydrates in serum suggested that free sugars were consumed for energy. A significant decrease in glucose and alpha-d-glucosamine 1-phosphate showed that chitin synthesis exhausted glycogen, resulting in insufficient glucose supply. An increase in l-carnitine and acetylcarnitine, and a significant decrease in 15 fatty acyls and 8 glycerophosphocholines in serum indicated that carnitine shuttle was stimulated, and β-oxidation was upregulated postmolt. In addition, astaxanthin, ponasterone A, and riboflavin in serum were significantly decreased postmolt. Eleven potential metabolite biomarkers were identified for pubertal molt. Taken together, carbohydrates and lipids were possibly major energy reserves in pubertal molt. This study suggests that an increase in carbohydrate and lipid levels in crab feed may alleviate the effects of fasting during molt and improve farm productivity in mature female crabs.

## 1. Introduction

*Scylla paramamosain*, known as the mud crab, is one of the most commercially important crustaceans distributed along the coast of southern China and broader Indo-Pacific countries [1]. Due to its considerable size, rapid growth, delicious flavor, and high market price, *S. paramamosain* has been an extremely important aquacultural species since the 1990s [2]. Nowadays, the production of mud crab has reached 157,712 tons in China [3]. Furthermore, female crabs with mature ovaries sell for a substantial price premium because their ovaries are considered to be a delicacy, suggesting the considerable effect of reproductive-system development on aquaculture [4,5]. Therefore, the reproductive development of female mud crabs is crucial in the *S. paramamosain* aquacultural industry.

Crustaceans are characterized by a rigid exoskeleton, which must periodically be shed (molted) for growth and reproduction [6]. The molting cycle comprises four major stages, ecdysis (molting), postmolt, intermolt, and premolt. Before pubertal molt (or terminal molt), the mud crab grows quickly via frequent molting (every 5–30 days, observation results). In pubertal molt, besides growth, gonad maturation is initiated, and mating promptly happens at the end of the pubertal molt, when the female is in the soft-shell condition [7]. Therefore, pubertal molt is not only a growth process, but essential for the copulation and reproductive development of the female mud crab.

Through molting cycles, crabs achieve saltatory changes or increments in growth. However, many researchers found a high incidence of molt death syndrome, which refers to death in a partially molted condition or suddenly after molting [8]. Although the cause of this problem is not fully understood, it is believed to be associated with inappropriate nutrition [8,9]. For example, the absence of phospholipids in their diet showed a negative effect for *Homarus americanus* and a resulting molt death syndrome [8]. Teshima et al., 1986 also reported that almost all *Marsupenaeus japonicus* larvae fed with phospholipid-deficient diet could not progress to zoeal 2 stage [10]. The nutritional requirements during ecdysis could be vital for the survival of crustaceans postmolt. Moreover, crustaceans naturally have fasting periods during molting. Feeding stops completely during molting, and begins again when the animal has a rigid enough exoskeleton to support its body weight and handle food [11,12]. No food intake (fasting) can lead to a severe deficiency of nutrients during molting [13].

Few studies are devoted to the influence of nutrition on molt of crustaceans [14]. Nguyen et al., 2014 reported that cumulative molts were clearly affected by the voluntary feed or energy intake levels, suggesting that molting was triggered when the energy threshold became sufficient [15]. A reduction in food supply might extend the intermolt period beyond its normal length [16]. Molting requires a significant amount of energy [17]. Catacutan., 2002 reported that the intermolt period of crabs was not shortened at elevated protein levels, suggesting that high protein level alone failed to promote molting [18]. Reports about preferential energy supply under fasting in crustaceans are contrasting [13]. Protein was first proposed to be the primary source of energy. Moreover, a series of studies on *Penaeus japonicus*, *Penaeus duorarum*, and *Crangon crangon* indicated that glycogen stores were rapidly depleted, lipids progressively diminished, and proteins were subsequently mobilized but more slowly [13]. Furthermore, the metabolic requirements of crustaceans in fasting appear to be species-specific. Protein was reported to be the primary energy supply in *Hemigrapsus nudus* and *Penaeus esculentus* [19,20]. Lipids are recruited as the preferential energy supply in *Niphargus virei* and *Niphargus rhenorhodanensis* [21]. Carbohydrates are the preferred energy supply in *Calanus finmarchicus* and *Palaemonetes argentinus* [22,23]. In addition, energy metabolism in fasting may vary depending on development stage. In the phyllosoma larvae of *Jasus edwardsii* starved for 6–11 days, lipids were the main energy source during food deprivation on late stages of development, while protein catabolism may be more important in stage I [24]. However, Johnston et al., 2004 reported that protease or lipase activities in phyllosoma larvae were found to be significantly increased or decreased in stages I and IV, respectively, suggesting that protein catabolism provided energy during food deprivation, while lipids may be spared during fasting [25]. Thus, differences in energy-source usage during fasting showed high variability, and this makes it too difficult to assume a standard metabolic profile [26].

Since previous studies of energy-reserve requirements generally focused on the molting of larval or juvenile crustaceans [13,25], little information is known on energy requirements in pubertal molting. Pubertal molting initiates the copulation and reproductive development of female mud crabs, has multiple implications for life history traits, and possibly demands different metabolic requirements than those of related decapod taxa or development stages. Moreover, reproduction is expensive in terms of energy expenditure, the requirements in pubertal molting are very demanding, and resources are tightly partitioned among survival, growth, and reproduction [27,28]. Hematological metabolites can reflect dietary nutrients, and they are useful indicators of nutritional state, molting, and reproductive variations [29]. A metabolomics approach focuses on low-weight molecules (molecular weight < 1000 Da), which are the end products of cellular regulatory processes [30]. In the present study, an investigation was performed to elucidate the changes of metabolites in serum in pre- and post-pubertal molt female crabs via a metabolomics approach. This work provides the basis to fulfill energy-reserve requirements during pubertal molting, and may lead to a marked improvement in the health and productivity of mature female crabs in the aquacultural industry.

## 2. Results

### 2.1. Overview of Metabolomic Profiles from Pre- and Post-Pubertal Molt Groups

**Figure 1:**
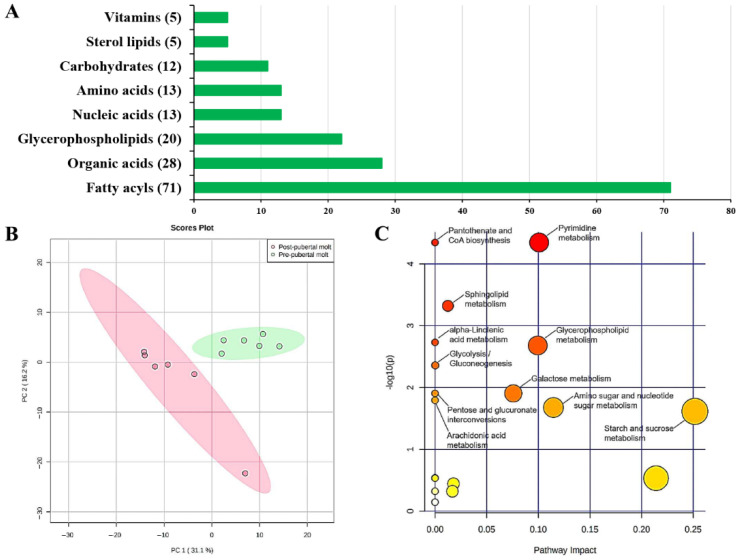
(**A**) Major metabolite classification, (**B**) PCA score plot, and (**C**) metabolite pathway analysis (MetPA) for metabolomic profiles in serum in the pre- and post-pubertal molt groups of *S. paramamosain*.

In this study, a common set of 338 metabolites were identified in the serum metabolomic profiles from pre- and post-pubertal molt groups. Metabolite enrichment analysis identified the first eight enriched metabolite categories in serum, namely, fatty acyls (71 metabolites), organic acids (28 metabolites), glycerophospholipids (20 metabolites), nucleic acids (13 metabolites), amino acids (13 metabolites), carbohydrates (12 metabolites), sterol lipids (5 metabolites), and vitamins (5 metabolites) (Figure 1A). In the multivariate analysis of metabolomic profiles, PCA score plot showed significant discrimination between the pre- and post-pubertal molt groups (Figure 1B). For effective clustering, oPLS-DA was conducted, and validated using a permutation test of training sets of 1000 times and prediction error of independent test sets. The permutation test demonstrated that the oPLS-DA model had good descriptive ability (R^2^Y = 99.6%) and high predictive ability (Q^2^ = 89.4%). On this basis, VIP scores were calculated and used in the identification of significantly modulated metabolites during pubertal molt (Table 1).

### 2.2. Significantly Changed Metabolites and Metabolite Pathway Analysis (MetPA)

Compared with the pre-pubertal molt group, 60 metabolites in the post-pubertal molt group were identified to be significantly changed (Table 1). Among these metabolites, 8 metabolites were upregulated and 52 metabolites were downregulated (Table 1). MetPA of the significantly changed metabolites revealed that 11 pathways were significantly enriched after pubertal molt (Figure 1C), which were separated into four KEGG pathway classes: carbohydrate metabolism (5 pathways, glycolysis or gluconeogenesis; galactose metabolism; pentose and glucuronate interconversions; amino sugar and nucleotide sugar metabolism; starch and sucrose metabolism), lipid metabolism (4 pathways, glycerophospholipid metabolism, arachidonic acid metabolism, sphingolipid metabolism, alpha-linolenic acid metabolism), nucleotide metabolism (pyrimidine metabolism), and the metabolism of cofactors and vitamins (pantothenate and CoA biosynthesis) (Figure 1C).

### 2.3. Carbohydrate Metabolites in Serum

**Figure 2:**
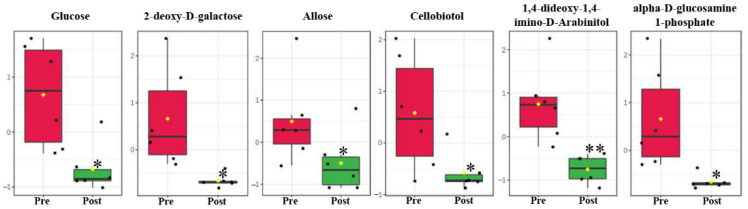
Changed carbohydrates in serum between pre- and post-pubertal molting groups. Values are means and standard deviations. Pre, pre-pubertal molt group. Post, post-pubertal molt group. Asterisks * and ** denote significant difference at the *p* < 0.05 and *p* < 0.01 levels, respectively.

In this study, 12 carbohydrates were identified in serum. Compared with the prepubertal molt group, 6 carbohydrates were recognized to be significantly changed postmolt, namely, glucose, 2-deoxy-d-galactose, allose, cellobiotol, 1,4-dideoxy-1,4-imino-d-arabinitol, and alpha-d-glucosamine 1-phosphate (Figure 2, Table 1).

### 2.4. Lipid Metabolites in Serum

Compared with the pre-pubertal molt group, 101 lipids in serum were identified via a metabolomics approach, including 71 fatty acyls (fatty acids) and 20 glycerophospholipids. Among all these lipids, 27 lipids in serum were significantly changed post-pubertal molt (Table 1), and were divided into four categories of LIPID MAPS database (https://www.lipidmaps.org/ (accessed on 16 March 2021)): glycerophospholipids (9 compounds), fatty acids (15 compounds), prenol lipids (astaxanthin), sterol lipids (ponasterone A), and sphingolipids (phytosphingosine). Nine glycerophospholipids were all glycerophosphocholines (GP01). Fifteen fatty acids were assigned to five subclasses as follows: fatty acids and conjugates (FA01, 8 fatty acids), eicosanoids (FA03, 4 fatty acids), docosanoids (FA04, 4-HDoHE), fatty alcohols (FA05, 1-hexadecanol), and fatty amides (FA08, stearamide). Astaxanthin, ponasterone A, and phytosphingosine were isoprenoids (PR01), sterols (ST01), and sphingoid bases (SP01), respectively (Table 1).

**Figure 3:**
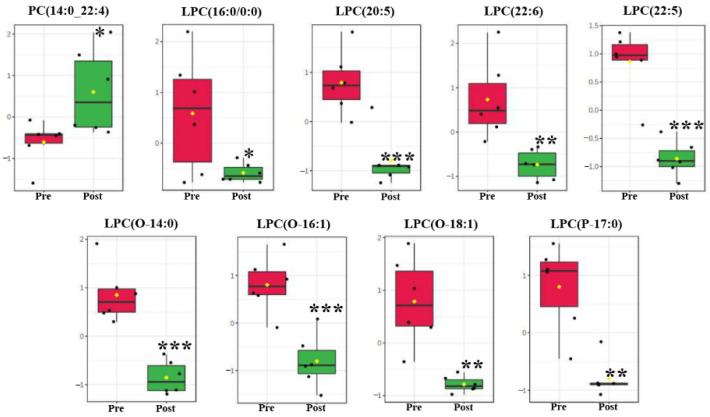
Significantly changed glycerophosphocholines between pre- and post-pubertal molting groups. Values are mean and standard deviation. Pre, pre-pubertal molt group. Post, post-pubertal molt group. Asterisks *, **, and *** denote significant difference at the *p* < 0.05, *p* < 0.01, and *p* < 0.001 levels, respectively.

**Figure 4:**
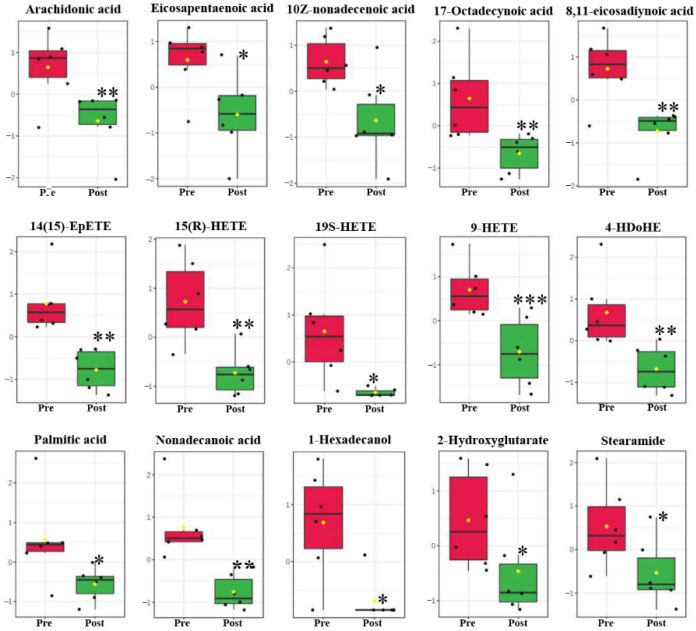
Significantly changed fatty acids between pre- and post-pubertal molting groups. First 10 fatty acids are unsaturated fatty acids. Values are mean and standard deviation. Pre, pre-pubertal molt group. Post, post-pubertal molt group. Asterisks *, **, and *** denote significant difference at the *p* < 0.05, *p* < 0.01, and *p* < 0.001 levels, respectively.

PC(14:0_22:4) was diacylglycerophosphocholine (GP0101) and significantly increased in content post-pubertal molt (Figure 3). The other eight glycerophosphocholines were monoacylglycerophosphocholines (GP0105) and significantly decreased in level postmolt, namely, LPC(16:0/0:0), LPC(20:5), LPC(22:6), LPC(22:5), LPC(O-14:0), LPC(O-16:1), LPC(O-18:1), and LPC(P-17:0) (Figure 3; Table 1). Furthermore, six more glycerophospholipids were recognized to be monoacylglycerophosphoethanolamine (GP0205), and none were significantly changed during pubertal molting (Table S1). All 15 fatty acids in serum were significantly decreased in abundance postmolt, and 10 of them were unsaturated fatty acids, including arachidonic acid (AA), eicosapentaenoic acid (EPA), 10Z-nonadecenoic acid, 17-octadecynoic acid, 8,11-eicosadiynoic acid, 14(15)-EpETE, 15(R)-HETE, 19S-HETE, 9-HETE, and 4-HDoHE (a hydroxy docosahexaenoic acid, hydroxy DHA) (Figure 4, Table 1).

### 2.5. Vitamin, Hormone Analog and Carnitine Shuttle

**Figure 5:**
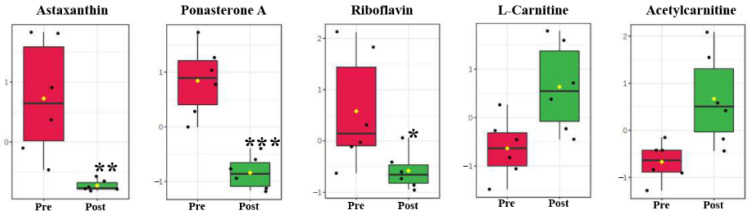
Change in astaxanthin, ponasterone A, riboflavin, l-carnitine, and acetylcarnitine between pre- and post-pubertal molting groups. Values are mean and standard deviation. Pre, pre-pubertal molt group. Post, post-pubertal molt group. Asterisks *, **, and *** denote significant difference at the *p* < 0.05, *p* < 0.01, and *p* < 0.001 levels, respectively.

Compared with the pre-pubertal molt group, astaxanthin, ponasterone A, and riboflavin (vitamin B2) were identified to be significantly changed postmolt (Figure 5). l-carnitine and acetylcarnitine, which are involved in the carnitine shuttle, were both increased in level postmolt (Figure 5).

### 2.6. Receiver Operating Characteristics (ROC) Analysis

In the present study, multivariate ROC curves from all models based on cross-validation performance indicated that the ROC model with 25 features (AUC = 0.965, CI = 0.75–1) reached the best predictive accuracy (95%). On the basis of this model, a potential biomarker panel containing 11 significantly changed metabolites with 3 upregulated and 8 downregulated metabolites postmolt was identified to be the best potential predictive biomarker for the pubertal molt of *S. paramamosain* (Table 2).

## 3. Discussion

In the present study, 338 metabolites were identified from serum samples in pre- and post-pubertal molt groups via a metabolomics approach. In total, 60 metabolites were recognized to be significantly changed postmolt, with 8 upregulated and 52 downregulated metabolites. Of the significantly changed metabolites, 27 and 6 were lipids and carbohydrates, respectively. Two nucleic acids (uracil, guanine), one amino acid (tryptophan), and one vitamin (riboflavin) were found to be significantly changed postmolt. Among all these remarkably changed metabolites, 26 lipids, 6 carbohydrates, guanine, tryptophan, and riboflavin were remarkably decreased in level postmolt. Only PC(14:0_22:4) and uracil were significantly increased in content. Therefore, lipids and carbohydrates were possibly major modulated metabolite classes in serum during the pubertal molt of *S. paramamosain.*

For most crustaceans, molting is a natural process for growth or reproduction, and a period of starvation (fasting) occurs [26]. Molting requires a significant amount of energy, and death follows if not satisfied [17]. Molt death syndrome has hampered the development of the crab-farming industry, especially in the cultivation of mature female crabs. However, information related to the dynamics of energy-reserve utilization during ecdysis is limited. Though previous studies propose that the main energy source for crustaceans is proteins [31], numerous recent reports indicate that the energy supply in short-term starvation conditions, which is similar to fasting during molting, is mainly and firstly carbohydrates and lipids [26,32]. Our study was in accordance with previous reports. Compared with the pre-pubertal molt group, 6 carbohydrates and 27 lipids in serum were significantly decreased, but few amino acids were remarkably influenced postmolt.

The remarkable decrease in free sugars, including glucose, suggested that free sugars could be directly used as energy supply in pubertal molting. Glucose usually represents the main circulating carbohydrate in crustaceans, which could be correlated to total body carbohydrate stores. The significant decrease in glucose in serum might indicate that glucose could be the first energy source consumed in pubertal molt. Glucose in crustaceans comes from two main sources: the direct absorption of dietary glucose and glycogen from hepatopancreas [13]. Since fasting happens in the pubertal molting process, glycogen in hepatopancreas could be the main source of free glucose. However, glycogen (carbohydrate) reserves in crustaceans are generally limited. Glycogen is both a glucose supply and an important precursor for chitin synthesis [26]. In this study, the significant decrease in alpha-d-glucosamine 1-phosphate in serum indicated that the synthesis of chitin was stimulated, which is the main molecular backbone in the exoskeleton (Figure 2). Therefore, the promoted chitin synthesis could have competitively consumed large amounts of glycogen, which resulted in an insufficient supply of free glucose in serum postmolt. The decrease in simple sugars in serum suggested that the energy requirement in pubertal molt might not be satisfied by single carbohydrates.

Lipids in serum were obviously changed post-pubertal molt of significantly changed metabolites, 45% were lipids (27/60, Table 1), including 9 glycerophosphocholines and 15 fatty acyls (fatty acids). These lipids influence 36.36% (4/11) of significantly enriched pathways (Figure 1C). Lipids are major organic constituents of decapod crustaceans, and they are catabolized to provide energy for various processes such as growth, molting, and reproduction [33]. In crustaceans, the hepatopancreas is the central organ for lipid metabolism, including the digestion, storage, catabolism, and anabolism of lipids [34]. When fasting occurs in molting, crustaceans activate lipid and glycogen metabolism in the hepatopancreas, with lipid metabolism dominating [35,36]. In the present study, 16 glycerophosphocholines were identified in serum during the pubertal molting, including 9 significantly changed glycerophosphocholines (Table S1 and Table 1, Figure 3). Glycerophosphocholines are an important member of phospholipids, which are essential components of lipoproteins. Phospholipids serve as a source of essential fatty acids or energy, and enhance lipid emulsification and transport to improve lipid deposition and energy utilization [37,38]. Thus, significantly changed glycerophosphocholines might play vital roles in lipid transport and energy supply. All monoalkyl-/monoacyl-/1Z-alkenyl-glycerophosphocholines in serum were decreased in content postmolt, including eight significantly changed glycerophosphocholines. Meanwhile, the levels of dialkyl-/1-alkyl-glycerophosphocholines and 2-acyl-glycerophosphocholines were increased, with one remarkably modulated glycerophosphocholine (Table S1 and Table 1, Figure 3). Lipases from *Homarus americanus* were observed to cleave triacylglycerides (mainly used as energy reserves in crustaceans) into monoacylglycerols, diacylglycerols, and free fatty acids [39]. Monoglycerides and free fatty acids in circulation are either catabolized by β-oxidation or transported to other tissue or cells for storage or synthesis [40]. Thus, the significant decrease in monoalkyl-/monoacyl-/1Z-alkenyl-glycerophosphocholines in serum could have been a sign of energy supply from monoglyceride lipids. Moreover, the increase in l-carnitine and acetylcarnitine content in serum postmolt suggests that the carnitine shuttle is activated, in which monoglycerides and free fatty acids are transported into the mitochondria for degradation by β-oxidation. Glycerophosphocholines and free fatty acids in serum could be recruited for energy post-pubertal molt.

Fatty acids play vital roles in the development of marine crustaceans as sources of energy and structural components of membranes [41]. In this study, 10 of 15 significantly modulated fatty acids were unsaturated fatty acids (66.7%, the first 10 fatty acids in Figure 4), suggesting that unsaturated fatty acids are important for crabs in pubertal molting. Arachidonic acid (AA), eicosapentaenoic acid (EPA), and 4-HdoHE (a hydroxy DHA, the main mediators of DHA in body) were all obviously decreased in serum postmolt (Figure 4). As essential long-chain polyunsaturated fatty acids (lc-PUFA) for crustaceans, AA, EPA, and DHA play key roles in the survival and growth of crustaceans. AA and EPA are preferentially accumulated in adult daphnids and allocated to the ovaries in the late stages of oocyte maturation [42]. AA and EPA are precursors to eicosanoids, associated with immune function, reproduction, and ion transport [43]. EPA and DHA are necessary for crustacean molting and development [44]. Moreover, DHA constitutes almost half of the lipid content of high-density lipoproteins and very-high-density lipoproteins in aquatic animals, responsible for efficient lipid transport [40]. Though approximately 40% of fatty acid uptake is catabolized by β-oxidation, this was the highest with AA, EPA, and DHA in rainbow trout, and over one-third of EPA was catabolized by β-oxidation in *Penaeus esculentus* [40]. Besides as energy reserves, lc-PUFA such as AA, EPA, and 4-HdoHE play various functions in physiological processes, as pubertal molting induces multiple physiological changes, including molting, mating, and reproductive development, which may require various individual nutrients with multiple functions. Several metabolites of AA, for example, prostaglandin E2, are associated with egg-laying behavior in insects [45]. Therefore, the obvious decrease in AA, EPA, 4-HdoHE, and other fatty acids in serum was due to both energy synthesis via β-oxidation and lipid accumulation in other tissue. Further studies should be performed to elucidate the functions of these lc-PUFA in pubertal molting.

**Figure 6:**
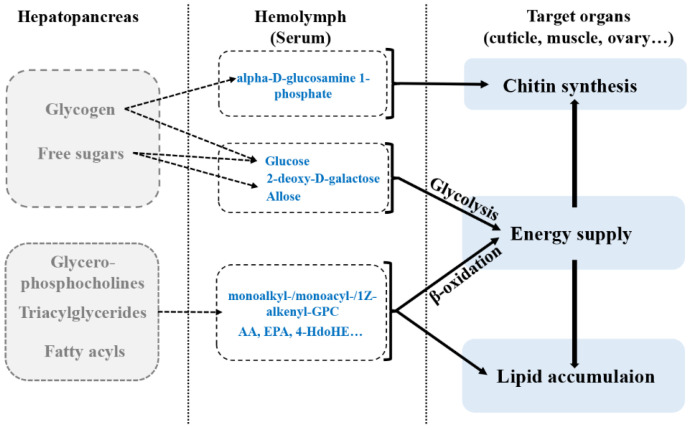
Metabolic profiles in serum of mud crabs post-pubertal molt. Blue, significantly decreased postmolt; gray, not detected.

**Figure 7:**
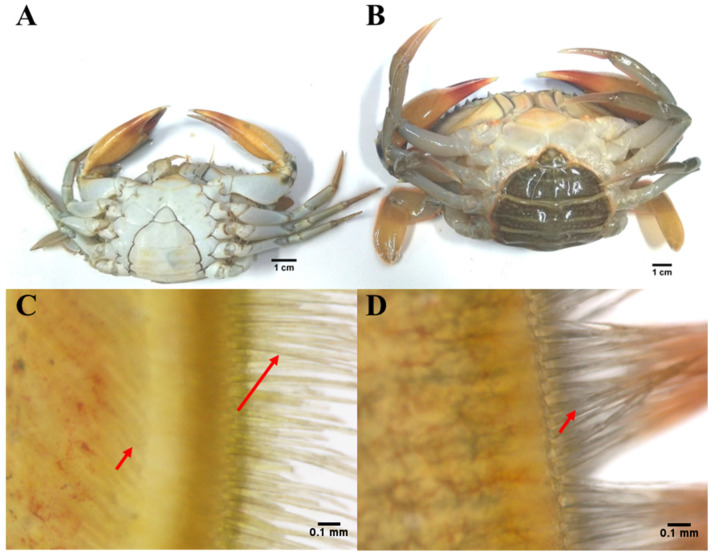
Experiment animals (*S. paramamosain*) in (**A**,**C**) pre- and (**B**,**D**) post-pubertal molt groups. (**A**) Crabs in pre-pubertal molt stage. (**B**) Crabs in post-pubertal molt stage. (**C**) Cuticle structure in swimming leg in pre-pubertal molt stage; long arrow, old setae; short arrow, new setae. (**D**) Cuticle structure in swimming leg in post-pubertal molt stage; short arrow, new setae.

Pubertal molting initiates the reproductive process of female mud crabs, which is essential for the cultivation of mature female crabs in the aquacultural industry. The metabolic profile in pubertal molt may be critical for the strategy of nutritional supply before molting. When fasting occurs during pubertal molting, the energy supply relies completely on inner energy reserves. Carbohydrates, especially free sugars in serum, are exhausted as a direct energy supply (Figure 6). However, the stimulated biosynthesis of chitin for cuticle construction postmolt may consume much glycogen, resulting in an insufficient supply of glucose (Figure 7). An insufficient glucose flux induces an energy gap, which requires other type of energy reserves for ATP synthesis. Lipids, including glycerophosphocholines and free fatty acids, are transported to the mitochondria via the carnitine shuttle and catabolized by β-oxidation. Thus, carbohydrates and lipids are major energy reserves during pubertal molting. Considering the multiple functions of fatty acids, including AA, EPA, and 4-HdoHE, free fatty acids could be accumulated in other tissue for reproductive development (Figure 6).

## 4. Materials and Methods

### 4.1. Experiment Animals and Serum Sampling

Mud crabs (*S. paramamosain,* female, 120 ± 10 g, at prepubertal molt stage, Figure 7A,C; male, 150 ± 10 g) were collected from a local fishing port in the city of Zhangzhou, Fujian province, China (23.93° N, 117.58° E). Each pair of crabs were reared in a water tank (80 cm × 60 cm × 40 cm) with filtered and aerated seawater at 27 °C, and fed with clams twice per day. Once pubertal molting had finished, female crabs (250 ± 20 g, Figure 7B,D) were captured for sampling. Female crabs at the pre-pubertal molt stage were sampled as control (Figure 7A,C). In total, 12 crabs in pre- and post-pubertal molt groups were collected for serum sampling, respectively. Hemolymph was collected aseptically (disinfected with 75% (*v*/*v*) ethanol) from the heart, and stored at room temperature for 1 h and overnight at 4 °C. After clotting, hemolymph was centrifuged at 16,000× *g* for 30 min, and supernatants (serum) were collected and stored at −80 °C until needed. All 12 serum samples were randomly divided into 6 parallel samples for metabolomics profiling.

### 4.2. Metabolite Extraction

Six parallel serum samples in pre- and post-pubertal molt groups were used in this study, respectively. Of each sample, 100 μL was extracted in 1000 μL of a precooled extraction solution (acetonitrile/methanol/water, 2:2:1 containing 1 μg/mL of internal standard, 2-chloro-l-phenylalanine). Each sample was vortexed for 30 s, homogenized at 45 Hz for 4 min, and sonicated for 5 min in an ice-water bath. After 3 homogenate and sonicate cycles, all samples were incubated at −20 °C for 1 h and centrifuged at 12,000 rpm and 4 °C for 15 min. The supernatant of each sample was transferred into a fresh tube and stored at −80 °C until analysis. The quality-control (QC) sample was prepared by mixing an equal aliquot of the supernatants from all samples.

### 4.3. Liquid Chromatography–Mass Spectrometry/Mass Spectrometry (LC-MS/MS) for Metabolomics Profiling

LC-MS/MS detection were performed with a UPLC HSS T3 column (2.1 mm × 100 mm, 1.8 μm, Waters) on a UHPLC system (1290, Agilent) coupled with Q Exactive MS (QE-MS, Orbitrap MS, Thermo, Waltham, MA, USA). The mobile phase A was 0.1% formic acid (CNW Technologies, Duesseldorf, Germany) in water for positive, 5 mmol/L ammonium acetate (CNW Technologies) in water for negative mode. The mobile phase B was acetonitrile (CNW Technologies). The elution gradient was set as follows: 0 min, 99% A + 1% B; 1 min, 99% A + 1% B; 8 min, 1% A + 99% B; 10 min, 1% A + 99% B; 10.1 min, 99% A + 1% B; and 12 min, 99% A + 1% B. The flow rate was 0.5 mL/min and the injection volume was 3 μL. QE-MS was used for acquiring MS/MS spectra on an information-dependent basis (IDA). In this mode, acquisition software (Xcalibur 4.0.27, Thermo, Waltham, MA, USA) continuously evaluated the full scan survey MS data as they were collected and triggered acquisition of MS/MS spectra depending on preselected criteria. ESI source conditions were set as follows: sheath gas flow rate was 45 Arb, aux gas flow rate was 15 Arb, and capillary temperature was 400 °C. The full MS resolution and MS/MS resolution were 70,000 and 17,500, respectively. The collision energy was set as 20/40/60 eV in the NCE model. The spray voltage was 4.0 kV (positive) or −3.6 kV (negative), respectively. LC-MS/MS detection and data analysis were performed by Biotree Biomedical Technology Co., Ltd. (Shanghai, China).

### 4.4. Gas Chromatograph/Time-of-Flight Mass Spectrometer (GC/TOF-MS) for Metabolomic Profiling

For GC/TOF-MS analysis, each sample prepared in Section 4.2 was evaporated in a vacuum concentrator, 40 μL of methoxyamination hydrochloride added (20 mg/mL in pyridine, TCI, Shanghai, China), incubated at 80 °C for 30 min, derivatized by 60 μL of N, O-bis (trimethylsilyl) trifluoroacetamide regent (1% trimethylchlorosilane, *v*/*v*, REGIS, Morton Grove, IL, USA) at 70 °C for 1.5 h, and then gradually cooled to room temperature. Furthermore, 5 μL of saturated fatty acid methyl ester (in chloroform, Dr. Ehrenstorfer, Augsburg, Germany) was added into the QC sample before analysis. All samples were analyzed by Agilent 7890 GC coupled with time-of-flight MS (GC-TOF/MS, Pegasus HT, Leco Corp., St. Joseph, MO, USA). The system utilized a DB-5MS capillary column (Agilent, Santa Clara, CA, USA), and 1 μL aliquot of each sample was injected in a splitless mode. Helium was used as carrier gas with 3 mL/min as front inlet purge flow and 1 mL/min as gas flow through the column. The initial temperature was maintained at 50 °C for 1 min and raised to 310 °C at a rate of 10 °C/min. The injection, transfer-line, and ion-source temperatures were 280, 280, and 250 °C, respectively. The energy was set as −70 eV in electron impact mode. Mass-spectrometry data were acquired in full-scan mode with the *m*/*z* range of 50–500 at a rate of 12.5 spectra per second after a solvent delay of 6.25 min. GC/TOF-MS detection and data analysis were performed in Biotree Biomedical Technology Co., Ltd. (Shanghai, China).

### 4.5. Metabolomic Data Preprocessing and Identification

The raw data from LC-MS/MS analysis were converted into the mzXML format using ProteoWizard, and processed by MAPS software (version 1.0, XCMS kernel), including peak detection, extraction, alignment, and integration. Preprocessing results generated a data matrix that consisted of retention time (RT), mass-to-charge ratio (*m*/*z*) values, and peak intensity. Metabolite identification was performed by matching MS/MS spectra using an in-house R program (developed on the basis of the previous study of Stein and Scott, 1994) against the Inhouse MS2 commercial database (Biotree, Shanghai, China) [46]. Raw data from GC-TOF-MS analysis, including peak extraction, baseline adjustment, deconvolution, alignment, and integration, were analyzed with Chroma TOF (version 4.3x, LECO) software. The LECO-Fiehn Rtx5 database was used for metabolite identification by matching mass spectrum and retention-time indices. The peaks detected in less than half of QC samples or RSD > 30% in QC samples were removed. To obtain a full page of all metabolites detected in this study, all identified metabolic data obtained from the positive and negative modes of LC-MS/MS and GC-TOF-MS were merged into a single file. Duplicate metabolites were screened on the basis of their scores, calculated using inhouse algorithms, and the metabolites with the highest scores were kept in this combined file (Biotree Biomedical Technology Co., Ltd., Shanghai, China).

### 4.6. Statistical Analyses

Metabolomics profiles were statistically analyzed via online analysis software MetaboAnalyst 5.0 (http://www.metaboanalyst.ca (accessed on 3 March 2021)) [47]. Before statistical analysis, metabolomic data were normalized by median and autoscaling. Multivariate principal-component analysis (PCA) and orthogonal projection to latent-structures discriminant analysis (oPLS-DA) were performed to assess discrimination between pre- and post-pubertal molt groups. The permutation test (1000 times) was performed to validate the quality of oPLS-DA model. The variable importance in the projection (VIP) of the first principal component of oPLS-DA was calculated to explain the importance of different metabolites. Two selection criteria were used to propose significantly changed metabolites after pubertal molt: (1) statistical significance (*p* value < 0.05, Student’s *t* test) and (2) VIP score > 1.

Metabolite pathway analysis (MetPA) was performed on the basis of pathway-associated metabolite sets [46]. Enrichment method, topology analysis, and pathway library were set as global test, relative-betweenness centrality, and *Drosophila melanogaster* (fruit fly, KEGG) [48].

Receiver operating characteristics (ROC) and areas under the ROC curve (AUC) were calculated to explore the discriminative capability of different metabolites via MetaboAnalyst 5.0. Multivariate exploratory ROC analysis, including feature selection, model building, and performance evaluation, were generated by Monte Carlo cross-validation (MCCV) [47]. Partial least-squares discriminant analysis (PLS-DA) for the classification and PLS-DA built-in for the feature ranking method were generated as a multivariate algorithm to perform biomarker identification.

## 5. Conclusions

In the present study, metabolomic profiles in the serum of female mud crabs in pre- and post-pubertal molting groups were investigated. Compared with the pre-pubertal molting group, carbohydrate and lipid metabolisms were remarkably affected after molting. Free sugars, especially glucose, were exhausted for energy supply postmolt. Chitin synthesis might consume large amounts of glycogen and result in insufficient glucose flux in serum. Lipids, including glycerophosphocholines and free fatty acids, were catabolized via β-oxidation for energy synthesis. In 15 obviously changed fatty acids, 10 were unsaturated fatty acids, suggesting that unsaturated fatty acids might play key roles in pubertal molting. Astaxanthin, ponasterone A, and riboflavin were significantly changed in pubertal molting. In conclusion, carbohydrates and lipids are major energy reserves consumed in pubertal molting. The present study provides a comprehensive perspective on the key energy metabolites in the serum during pubertal molting. On this basis, further study is valuable for developing a proper strategy of nutrition supply for female mud crabs before pubertal molting in the aquacultural industry.

## Figures and Tables

**Table 1:** Significantly changed metabolites between pre- and post-pubertal molt groups.

Category	Subclass	Compound Name	Score ^1^	Rt ^2^	Mz ^3^	Platform ^4^	Post-Pubertal Molt		Pre-Pubertal Molt		VIP Score	*p* Value	Fold Change (Post/Pre)
							Mean	Std	Mean	Std			
Lipids	Glycerophospholipids	PC(14:0_22:4)	0.967	513.5	782.56	POS	1.00 × 10^−4^	3.02 × 10^−5^	6.17 × 10^−5^	2.67 × 10^−5^	1.146	0.04116	1.625
		LPC(16:0/0:0)	0.903	450.7	496.34	POS	2.63 × 10^−5^	9.16 × 10^−6^	7.79 × 10^−5^	4.75 × 10^−5^	1.556	0.04460	0.338
		LPC(20:5)	0.724	410.9	542.32	POS	8.55 × 10^−6^	4.12 × 10^−6^	2.86 × 10^−5^	6.08 × 10^−6^	2.235	0.00005	0.299
		LPC(22:6)	0.793	430.1	568.34	POS	1.42 × 10^−5^	6.30 × 10^−6^	5.18 × 10^−5^	1.84 × 10^−5^	2.098	0.00296	0.275
		LPC(22:5)	0.864	439.7	570.35	POS	1.84 × 10^−6^	1.02 × 10^−6^	7.87 × 10^−6^	1.93 × 10^−6^	2.203	0.00005	0.233
		LPC(O-14:0)	0.644	419.4	454.33	POS	2.19 × 10^−5^	6.68 × 10^−6^	7.49 × 10^−5^	1.99 × 10^−5^	2.284	0.00076	0.293
		LPC(O-16:1)	0.795	444.3	480.34	POS	9.69 × 10^−6^	4.13 × 10^−6^	2.90 × 10^−5^	7.15 × 10^−6^	2.110	0.00019	0.334
		LPC(O-18:1)	0.559	509.1	508.37	POS	4.74 × 10^−6^	2.34 × 10^−6^	4.32 × 10^−5^	2.19 × 10^−5^	2.252	0.00745	0.110
		LPC(P-17:0)	0.749	480.4	494.36	POS	7.76 × 10^−6^	5.11 × 10^−6^	4.73 × 10^−5^	1.85 × 10^−5^	2.210	0.00262	0.164
	Fatty acyls	Palmitic acid	0.932	1162.0	117.00	GC	2.01 × 10^−5^	9.46 × 10^−6^	4.56 × 10^−5^	2.48 × 10^−5^	1.526	0.04039	0.441
		Nonadecanoic acid	0.996	617.2	297.28	NEG	9.99 × 10^−5^	6.45 × 10^−5^	2.86 × 10^−4^	9.73 × 10^−5^	1.932	0.00298	0.350
		Arachidonic acid	0.995	504.5	303.23	NEG	1.45 × 10^−3^	5.39 × 10^−4^	2.95 × 10^−3^	7.91 × 10^−4^	1.669	0.00318	0.490
		10Z-nonadecenoic acid	0.994	572.9	295.26	NEG	2.74 × 10^−4^	1.73 × 10^−4^	4.87 × 10^−4^	7.96 × 10^−5^	1.690	0.02093	0.563
		17-Octadecynoic acid	0.999	498.9	279.23	NEG	8.43 × 10^−6^	7.46 × 10^−7^	1.33 × 10^−5^	2.97 × 10^−6^	2.025	0.00930	0.635
		8,11-eicosadiynoic acid	0.908	523.9	303.23	NEG	5.89 × 10^−5^	2.11 × 10^−5^	1.23 × 10^−4^	2.81 × 10^−5^	1.906	0.00120	0.478
		Eicosapentaenoic acid	0.984	474.7	301.22	NEG	1.05 × 10^−3^	7.09 × 10^−4^	1.99 × 10^−3^	5.09 × 10^−4^	1.526	0.02503	0.529
		2-Hydroxyglutarate	0.997	26.8	147.03	NEG	7.26 × 10^−5^	6.35 × 10^−5^	1.68 × 10^−4^	8.01 × 10^−5^	1.650	0.04580	0.433
		14(15)-EpETE	0.868	382.7	317.21	NEG	6.15 × 10^−6^	4.62 × 10^−6^	2.83 × 10^−5^	1.09 × 10^−5^	1.724	0.00102	0.217
		15(R)-HETE	0.891	467.8	303.23	POS	4.14 × 10^−6^	4.37 × 10^−6^	2.22 × 10^−5^	9.47 × 10^−6^	1.404	0.00174	0.187
		19S-HETE	0.817	516.2	319.23	NEG	2.78 × 10^−7^	7.00 × 10^−6^	8.24 × 10^−6^	3.77 × 10^−7^	1.889	0.03851	0.034
		9-HETE	0.713	406.9	319.23	NEG	1.02 × 10^−5^	7.12 × 10^−6^	3.02 × 10^−5^	7.94 × 10^−6^	1.761	0.00100	0.338
		4-HDoHE	0.680	401.8	343.23	NEG	5.46 × 10^−6^	4.53 × 10^−6^	2.22 × 10^−5^	1.10 × 10^−5^	1.627	0.00627	0.246
		1-Hexadecanol	0.724	1111.7	75.00	GC	1.38 × 10^−7^	3.36 × 10^−7^	1.32 × 10^−6^	8.63 × 10^−7^	1.645	0.01063	0.104
		Stearamide	0.997	585.1	284.29	POS	2.58 × 10^−5^	1.57 × 10^−5^	5.16 × 10^−5^	2.16 × 10^−5^	1.625	0.03936	0.500
	Prenol Lipids	Astaxanthin	0.827	590.3	597.39	POS	2.60 × 10^−6^	3.11 × 10^−6^	4.48 × 10^−5^	2.55 × 10^−5^	2.038	0.00944	0.058
	Sterol Lipids	Ponasterone A	0.831	317.4	465.32	POS	6.21 × 10^−6^	2.09 × 10^−6^	2.18 × 10^−5^	6.15 × 10^−6^	2.225	0.00098	0.284
	Sphingolipids	Phytosphingosine	0.689	361.7	318.30	POS	3.96 × 10^−5^	7.79 × 10^−6^	3.15 × 10^−5^	2.44 × 10^−6^	1.507	0.04973	1.260
Carbohydrates		Glucose	0.944	1081.6	73.00	GC	5.32 × 10^−5^	6.36 × 10^−5^	2.97 × 10^−4^	1.59 × 10^−4^	1.921	0.00590	0.179
		2-Deoxy-d-galactose	0.579	1007.3	103.00	GC	9.54 × 10^−7^	1.08 × 10^−6^	9.63 × 10^−6^	7.41 × 10^−6^	1.525	0.03465	0.099
		Allose	0.497	915.5	117.00	GC	3.70 × 10^−8^	4.14 × 10^−8^	1.41 × 10^−7^	1.01 × 10^−7^	1.477	0.04207	0.263
		Cellobiotol	0.748	1513.0	73.00	GC	1.65 × 10^−6^	1.86 × 10^−6^	9.33 × 10^−6^	7.32 × 10^−6^	1.694	0.04945	0.176
		1,4-Dideoxy-1,4-imino-d-arabinitol	0.898	46.3	134.08	POS	1.07 × 10^−5^	6.83 × 10^−6^	3.69 × 10^−5^	1.25 × 10^−5^	1.935	0.00114	0.290
		alpha-d-Glucosamine 1-phosphate	0.469	1016.4	103.00	GC	1.46 × 10^−6^	1.78 × 10^−6^	1.49 × 10^−5^	1.16 × 10^−5^	2.001	0.03534	0.098
Vitamin B2		Riboflavin	0.852	230.3	377.14	POS	7.43 × 10^−6^	3.15 × 10^−6^	2.39 × 10^−5^	1.46 × 10^−5^	1.809	0.03828	0.310
Neurotransmitter		Acetylcholine	0.981	35.9	146.12	POS	1.11 × 10^−3^	2.07 × 10^−4^	5.99 × 10^−4^	2.30 × 10^−4^	1.861	0.00248	1.845
Nucleic acids		Uracil	0.997	59.1	113.03	POS	1.76 × 10^−4^	5.35 × 10^−5^	9.86 × 10^−5^	2.42 × 10^−5^	1.885	0.00891	1.787
		Guanine	0.990	74.5	150.04	NEG	2.29 × 10^−5^	9.93 × 10^−6^	5.15 × 10^−5^	1.56 × 10^−5^	1.875	0.00356	0.445
Amino acid		Tryptophan	0.736	193.8	203.08	NEG	2.27 × 10^−4^	1.82 × 10^−4^	8.34 × 10^−4^	4.99 × 10^−4^	1.778	0.02970	0.272
		trans-2-Hydroxycinnamic acid	0.393	1026.2	102.00	GC	4.40 × 10^−10^	7.60 × 10^−12^	9.90 × 10^−8^	7.66 × 10^−8^	2.062	0.02538	0.004
		Taxifolin	0.472	26.7	303.05	NEG	2.49 × 10^−5^	1.80 × 10^−5^	8.88 × 10^−5^	2.91 × 10^−5^	2.079	0.00102	0.280
		Sulfoacetic acid	0.739	26.6	138.97	NEG	5.96 × 10^−4^	2.86 × 10^−4^	2.55 × 10^−5^	2.51 × 10^−5^	2.219	0.00444	23.357
		Plumbagin	0.560	549.7	187.04	NEG	1.84 × 10^−4^	9.72 × 10^−5^	4.55 × 10^−4^	1.69 × 10^−4^	1.705	0.00676	0.404
		Phytomonic acid	0.983	605.0	295.26	NEG	1.85 × 10^−5^	1.47 × 10^−5^	5.24 × 10^−5^	1.75 × 10^−5^	1.788	0.00459	0.353
		Pelargonate	0.999	557.1	157.12	NEG	2.68 × 10^−4^	8.10 × 10^−5^	4.32 × 10^−4^	1.41 × 10^−4^	1.655	0.03322	0.621
		Oleic acid ethyl ester	0.997	600.3	309.28	NEG	5.67 × 10^−4^	4.21 × 10^−4^	1.05 × 10^−3^	2.32 × 10^−4^	1.583	0.03257	0.538
		Mytilin A	0.903	46.5	333.13	POS	3.66 × 10^−6^	2.82 × 10^−6^	9.31 × 10^−5^	7.59 × 10^−5^	2.084	0.03433	0.039
		Methyl heptadecanoic acid	0.997	574.2	283.26	NEG	9.41 × 10^−5^	1.38 × 10^−5^	1.19 × 10^−4^	2.12 × 10^−5^	1.442	0.04009	0.794
		Isoxanthopterin	0.422	1213.8	73.00	GC	1.14 × 10^−6^	2.13 × 10^−7^	7.95 × 10^−7^	2.05 × 10^−7^	1.643	0.01774	1.430
		Isokobusone	0.821	457.4	221.15	NEG	2.55 × 10^−5^	2.45 × 10^−6^	2.95 × 10^−5^	2.36 × 10^−6^	1.629	0.01791	0.867
		Idazoxan	0.966	204.3	205.10	POS	1.26 × 10^−3^	1.01 × 10^−3^	4.53 × 10^−3^	2.68 × 10^−3^	1.771	0.01892	0.277
		Glutaraldehyde	0.203	527.6	100.00	GC	4.90 × 10^−7^	1.73 × 10^−7^	1.02 × 10^−6^	3.09 × 10^−7^	1.960	0.00422	0.479
		Glucoheptonic acid	0.581	1209.7	73.00	GC	9.49 × 10^−7^	4.74 × 10^−7^	1.96 × 10^−6^	3.79 × 10^−7^	2.031	0.00219	0.484
		Ethyldodecanoate	0.626	536.5	227.20	NEG	6.26 × 10^−5^	1.36 × 10^−5^	3.81 × 10^−5^	1.74 × 10^−5^	1.254	0.02164	1.643
		Diglycerol	0.566	986.0	292.00	GC	7.35 × 10^−8^	1.81 × 10^−8^	1.09 × 10^−7^	2.50 × 10^−8^	1.603	0.01831	0.675
		Di(2-ethylhexyl)phthalate	0.994	612.1	391.28	POS	6.79 × 10^−5^	1.32 × 10^−5^	5.32 × 10^−5^	3.63 × 10^−6^	1.602	0.04011	1.277
		Debromohymenialdisine	0.846	42.9	246.10	POS	3.89 × 10^−6^	7.01 × 10^−6^	5.95 × 10^−5^	3.48 × 10^−5^	2.124	0.01046	0.065
		5(Z),14(Z)-Eicosadienoic acid	0.999	558.9	307.26	NEG	5.17 × 10^−4^	2.53 × 10^−4^	1.01 × 10^−3^	1.58 × 10^−4^	1.822	0.00250	0.514
		4-Formyl indole	0.982	280.8	144.04	NEG	6.81 × 10^−5^	6.28 × 10^−6^	9.17 × 10^−5^	1.60 × 10^−5^	1.853	0.00721	0.743
		2-Butyne-1,4-diol	0.609	593.5	244.00	GC	5.34 × 10^−8^	8.26 × 10^−8^	2.06 × 10^−7^	1.03 × 10^−7^	1.306	0.01769	0.259
		1,5-Naphthalenediamine	0.878	204.3	159.09	POS	2.88 × 10^−5^	2.21 × 10^−5^	9.87 × 10^−5^	5.45 × 10^−5^	1.792	0.01549	0.291

^1^ Annotation score (AnnoScore = dotproduct1/sqrt(common), common = matched fragments number). ^2^ Retention time (second). ^3^ Mass-to-charge ratio value (measured value). ^4^ POS, HPLC positive mode; NEG, HPLC negative mode; GC, gas chromatography.

**Table 2:** Potential metabolite biomarkers for pubertal molt in serum of *S. paramamosain*.

Metabolite	AUC	*p* Value	Log_2_ Fold Change (Post/Pre)
Sulfoacetic acid	1	0.00012	4.53038
Uracil	1	0.00489	0.82525
Ethyldodecanoate	1	0.00531	0.80785
LPC(22:5)	1	0.00007	−0.57454
LPC(O–14:0)	1	0.00010	−1.65232
Ponasterone A	1	0.00017	−1.11677
LPC(O–18:1)	1	0.00103	−2.03488
14(15)–EpETE	1	0.00132	−1.44197
Nonadecanoic acid	1	0.00222	−1.67867
LPC(22:6)	1	0.00375	−1.59613
Astaxanthin	1	0.00432	−2.23112

## Data Availability

Data are available upon reasonable request. Additional data (beyond those included in the main text and Supplementary Materials) are available from the corresponding author upon request.

## Supplementary Materials

The following are available online at https://www.mdpi.com/article/10.3390/metabo11100651/s1. Table S1: identified glycerophospholipids in pre- and post-pubertal molt groups.
